# Donor human milk and structural brain development in very preterm infants

**DOI:** 10.1038/s41390-025-04539-3

**Published:** 2025-11-01

**Authors:** Katherine M. Ottolini, Sudeepta K. Basu, Julius Ngwa, Kushal Kapse, Jonathan Murnick, Kevin M. Cook, Yao Wu, Tameka T. Watson, Adre J. du Plessis, Catherine Limperopoulos, Nickie Andescavage

**Affiliations:** 1 Developing Brain Institute, Children’s National Hospital, Washington, DC USA; 2 Division of Neonatology, Children’s National Hospital, Washington, DC USA; 3 Department of Pediatrics, The George Washington University School of Medicine and Health Sciences, Washington, DC USA; 4 Division of Diagnostic Imaging and Radiology, Children’s National Hospital, Washington, DC USA; 5 Department of Radiology, The George Washington University School of Medicine and Health Sciences, Washington, DC USA; 6 Division of Prenatal and Transitional Pediatrics, Children’s National Hospital, Washington, DC USA

## Abstract

**Background:**

We investigated the impact of donor milk on structural brain development, relative to maternal milk feeding, in very preterm infants at term-equivalent age (TEA).

**Methods:**

Very preterm infants ( ≤32 weeks’ gestation) were enrolled in an observational study. Infants categorized as receiving primarily maternal milk (MOM), donor human milk (DHM), or preterm formula (PTF) based on cumulative feed volume underwent TEA brain magnetic resonance imaging for volumetric (coronal T2) and white matter (diffusion tensor imaging) development.

**Results:**

In this cohort of 152 infants (67 MOM, 44 DHM, 41 PTF), there were no significant differences in brain volumes between MOM and DHM. PTF demonstrated lower deep gray matter (β = –1.2, *p* = 0.024), brainstem (β = –0.4, *p* = 0.002) and total brain volumes (β = –17.2, *p* = 0.016) than MOM. Relative to MOM, DHM showed decreased white matter mean diffusivity in the pons (right: β = –0.04, *p* = 0.008; left: β = –0.06, *p* < 0.001); PTF had increased mean diffusivity in the corpus callosum (genu: β = 0.11, *p* = 0.007; splenium: β = 0.13, *p* = 0.007) and posterior limb of internal capsule (PLIC) (right: β = 0.06, *p* = 0.002; left: β = 0.06, *p* = 0.001) and decreased fractional anisotropy in the right PLIC (β = –0.02, *p* = 0.019).

**Conclusions:**

Brain volumes and overall white matter development were not significantly different between DHM and MOM, while PTF-fed infants demonstrated lower total and regional brain volumes and greater white matter microstructural alterations.

**Impact:**

- Feeding very preterm infants maternal milk is associated with improved *ex-utero* third trimester brain development compared to formula, but the implications of donor milk for structural brain development remain unknown.
- We performed brain magnetic resonance imaging at term-equivalent age to compare structural brain development between very preterm infants primarily fed either maternal milk, donor milk, or preterm formula.
- Brain volumes and white matter microstructure in primarily donor milk-fed very preterm infants more closely resembled that of maternal-milk fed infants than did infants fed formula, suggesting donor milk offers a potential advantage for structural brain development when maternal milk is unavailable.

## Introduction

The third trimester of pregnancy encompasses a critical neurodevelopmental window of exponential brain growth and complex neuro-programming that very preterm infants must undergo within the extrauterine environment.^1–3^ Developing amidst a barrage of noxious and stressful stimuli, the preterm *ex-utero* brain is particularly vulnerable to injury and dysmaturation accompanied by a high incidence of lifelong neurodevelopmental impairment.^4–7^ Maternal milk feeding during this *ex-utero* third trimester is associated with neurodevelopmental benefits through adolescence.^8–13^ Despite these well-established benefits, mothers of preterm infants often struggle to provide sufficient milk to meet nutritional demands due to a multitude of biological and psychosocial barriers.^14–16^

Nutritional supplementation with donor human milk is recommended for very preterm infants when maternal milk is unavailable, but it remains unclear whether the neuroprotective effects of preterm maternal milk extend to pasteurized donor milk containing decreased macronutrients and bioactive molecules.^17–21^ In two large randomized-controlled trials, neurodevelopmental outcomes at toddler age were similar in very preterm infants who received nutrient-fortified pasteurized donor human milk compared to preterm formula as either their primary diet or in supplementation to maternal milk.^22,23^ However, a limitation of traditional neurodevelopmental outcome studies is the time interval between in-hospital nutritional exposures and subsequent neurodevelopmental assessments, with the potential for additional confounding following hospital discharge. Neonatal quantitative brain magnetic resonance imaging (qMRI) provides a more proximate measure of the impact of nutritional exposures on *ex-utero* third trimester brain development through non-invasive, real-time assessments, revealing important biomarkers for later neurodevelopment.^24–27^

Observational studies using term-equivalent qMRI have linked preterm maternal milk exposure to improved total and tissue-specific brain growth, white matter development, and functional connectivity in a dose-dependent fashion compared to formula feeding.^9,12,28–34^ We previously reported a positive association between donor milk intake and structural brain development in a small subset of human milk-fed very preterm infants as part of an exploratory secondary analysis, but the effects of donor human milk on preterm brain development have not been specifically explored using qMRI.^30^ In this cohort study of very preterm infants receiving nutritional support with pasteurized donor human milk, mother’s own milk, or preterm formula, we aim to investigate the relationship between type of primary enteral nutrition and structural brain development at term-equivalent age. Using mother’s own milk as the reference standard, we hypothesize that term-equivalent qMRI will reveal greater differences in brain size and white matter maturation between preterm formula and maternal milk-fed infants than those supported with donor human milk.

## Methods

### Subjects

Preterm infants admitted to the level IV NICU at Children’s National Hospital (Washington, D.C.) from March 2012 through September 2022 were enrolled as part of a prospective, observational study of the antecedents and sequelae of prematurity-related brain injury.^30^ As a freestanding children’s hospital, all infants admitted to the NICU were out born. Infants born very preterm ( ≤32 weeks gestational age) and/or very low birth weight ( <1500 g) were eligible for inclusion if they were admitted within the first two weeks of life and received at least two weeks of enteral nutrition during NICU hospitalization. Infants with a known or suspected underlying genetic syndrome, metabolic disorder, or perinatal central nervous system infection were excluded from enrollment. Additionally, infants were excluded if either screening head ultrasound or subsequent MRI demonstrated structural brain malformations or significant injury (grade III intraventricular hemorrhage, periventricular hemorrhagic infarction, or cerebellar hemorrhage). This study was approved by the Children’s National Hospital Institutional Review Board, and informed, written consent was obtained from the parents of all participants. This study was conducted in accordance with the Strengthening the Reporting of Observational Studies in Epidemiology (STROBE) guidelines for observational cohort studies.

Infant race, ethnicity, and home address were self-reported; the remainder of demographics and clinical data were extracted from the medical record. Home address was used to calculate the overall Social Vulnerability Index (SVI), with indices ranging from 0 to 1 representing low to high vulnerability.^35^ Common comorbidities known to affect preterm nutritional advancement and neurodevelopment outcomes were also evaluated, including any diagnosis of infection (positive microbial test or ≥7 days of antibiotic treatment), systemic steroid treatment (hydrocortisone or dexamethasone), bronchopulmonary dysplasia (respiratory support administered at 36 weeks postmenstrual age),^36^ surgical necrotizing enterocolitis or spontaneous intestinal perforation (requiring laparotomy or drain placement), patent ductus arteriosus, retinopathy of prematurity (greater than or equal to stage 2), and intraventricular hemorrhage (Papile’s grading system). Weekly anthropometric measures (weight, length, head circumference) were also extracted from the medical record, with growth faltering defined as a decrease in weight z-score by >1 standard deviation from birth to term-equivalent age.^37,38^

### Nutrition

Daily nutritional intake (parenteral and enteral) was retrospectively collected from the electronic medical record. The parenteral nutrition protocol initiated protein upon NICU admission with 3 g/kg/day of amino acids and advanced the following day to a goal of 3.5-4 g/kg/day. Intravenous lipids were initiated at 1 g/kg/day within 24 h of NICU admission and advanced by 1 g/kg/day, as tolerated, to a goal of 3 g/kg/day.

A standardized feeding protocol for very preterm infants detailed specific parameters based on birth weight and feeding tolerance. Trophic enteral feeds were initiated at 10–20 ml/kg/day within 24 h of life or once the infant was deemed clinically stable by the medical team, followed by daily advancement by 20 ml/kg/day, as tolerated, to a goal feeding volume of 160 ml/kg/day. Growth faltering was managed by increasing the caloric density of feeds (i.e. via the provision of additional formula supplementation to achieve >24 kcal per ounce) or addition of modular macronutrient supplements (liquid protein, MCT oil), based upon the primary enteral nutrition type and infant’s growth velocity. Pasteurized donor human milk was available beginning in June 2014 and was provided with parental consent when maternal milk supply was insufficient until approximately 34 weeks corrected age, at which point infants were gradually transitioned to preterm formula. Maternal (unpasteurized) and donor human milk were both fortified in the same fashion with a liquid bovine-based human milk fortifier to assumed 22 kcal per ounce at a feeding volume of 80 ml/kg/day and 24 kcal per ounce at a feeding volume of 100 ml/kg/day. Daily enteral feeding volume was recorded from admission until discharge or TEA MRI, whichever was achieved sooner. Preterm infants were categorized by primary enteral nutrition type of mother’s own milk, donor human milk, or preterm formula ( >50% cumulative enteral feed volume).

### MRI acquisition and processing

Non-sedated brain MRI studies were performed at TEA on a 3 Tesla MRI scanner (Discovery MR750; General Electric Medical, Systems, Waukesha, Wisconsin) with an 8-channel receiver head coil approved for safety in neonates. Infants were immobilized using an InfantVacuum Immobilizer (Newmatic Medical, Caledonia, Michigan) and provided with double ear protection. The MRI acquisition protocol included structural imaging with T2 3D-cube and T1 3D-spoiled gradient recalled images (T2: 84 ms echo time, 2500 ms repetition time, field of view 13 cm, 1 mm slice thickness, 160 × 160 acquisition matrix; T1: 3.8 ms echo time, 6.7 ms repetition time, field of view 13 cm, 1 mm slice thickness, 700 ms inversion time, 160 × 160 acquisition matrix). DTI acquisition consisted of a single-shot, echoplanar sequence with 27 or 64 (High Angular Resolution Diffusion Imaging-HARDI) noncollinear direction diffusion gradients with an effective high b-value of 2500 s/mm^2^ (3 b = 0 s/mm^2^; HARDI 4 b = 0 s/mm^2^), 80 ms echo time, 8000 ms repetition time, field of view 200 × 200 mm, 3 mm slice thickness and no gap with a 128 × 128 acquisition matrix. Each MRI study was reviewed by an experienced pediatric neuroradiologist (JM) and assigned a Kidokoro score for white matter injury reflecting cystic lesions (score 0–4) or focal signal abnormalities (score 0–3) using T1 and T2 structural imaging.^39^

**Fig. 1:**
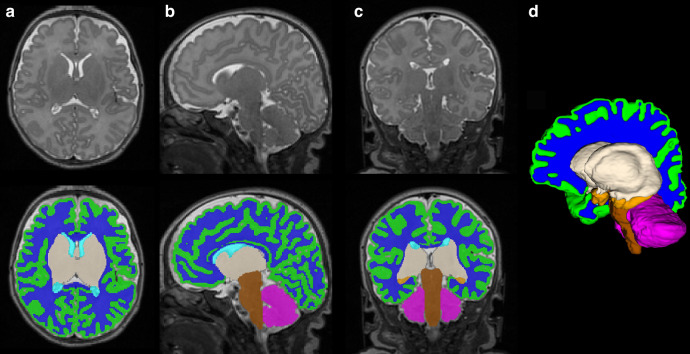
Term equivalent volumetric segmentation images. T2 brain MRI images with corresponding volumetric segmentation in the **a** axial, **b** sagittal, and **c** coronal planes with **d** 3D volumetric reconstruction. *Green*: cortical gray matter, *blue:* white matter, *gray*: deep gray matter, *tan*: amygdala-hippocampus, *pink*: cerebellum, *brown*: brainstem.

Volumetric segmentation was performed on coronal T2 Cube 3D images using a validated automated algorithm via the Draw-EM (Developing brain Region Annotation With Expectation-Maximization) package,^40^ with subsequent manual inspection and correction as needed by two investigators blinded to enteral nutrition type (KO and KK).^37^ Tissue-specific brain volumes were obtained for the cortical and deep gray matter, white matter, amygdala-hippocampus, cerebellum, and brainstem; total brain volume was calculated as the sum of all tissue-specific brain volumes for each infant (Fig. 1). DTI data were preprocessed based on a previously published pipeline, with cubic regions of interest (21-49mm^3^) manually placed by two investigators blinded to enteral nutrition type (KO and KK) using predefined anatomical landmarks in the corpus callosum (genu and splenium), posterior limb of internal capsule, and brainstem (pons).^41^ Fractional anisotropy (FA) and mean diffusivity (MD) values, measuring directionality and net diffusion of water molecules, respectively, were calculated for each region of interest. Inter- and intra-rater reliability measures for manually corrected MRI brain volumes and DTI region of interest placement were calculated based on a randomly selected subset of 35 patients using the intraclass correlation coefficient.

### Statistical analysis

Statistical analysis was performed using R Software (R Studio version 2023.12.1.402). Patient characteristics were reported using means and standard deviations (SD) or medians and interquartile ranges (IQRs) for normally and non-normally distributed continuous measures, respectively; categorical data were reported as frequencies (percentages). Univariate analyses using analysis of variance (ANOVA) or Kruskal-Wallis tests for continuous data and chi-square or Fisher’s exact tests for categorical data were conducted to identify patient characteristics that differed significantly based on enteral nutrition type. Generalized linear models adjusting for gestational age at birth and postmenstrual age at MRI were used to compare quantitative brain MRI measures (volume and DTI) based on enteral nutrition type. In these models, mother’s own milk was used as the reference enteral nutrition type against which donor human milk and preterm formula were compared. Additional models further incorporated infant sex and SVI as potential covariates. *P* values were two-tailed, with threshold *p* < 0.025 considered statistically significant to account for multiple comparisons, and confidence intervals set at 95%. Quantitative brain MRI measures (volume and DTI) between donor human milk and formula-fed infants were compared as a post-hoc analysis. A sensitivity analysis to assess nutritional exposure effect was performed between maternal and donor human milk-fed infants using a threshold of >70% of cumulative enteral nutrition.

## Results

**Fig. 2:**
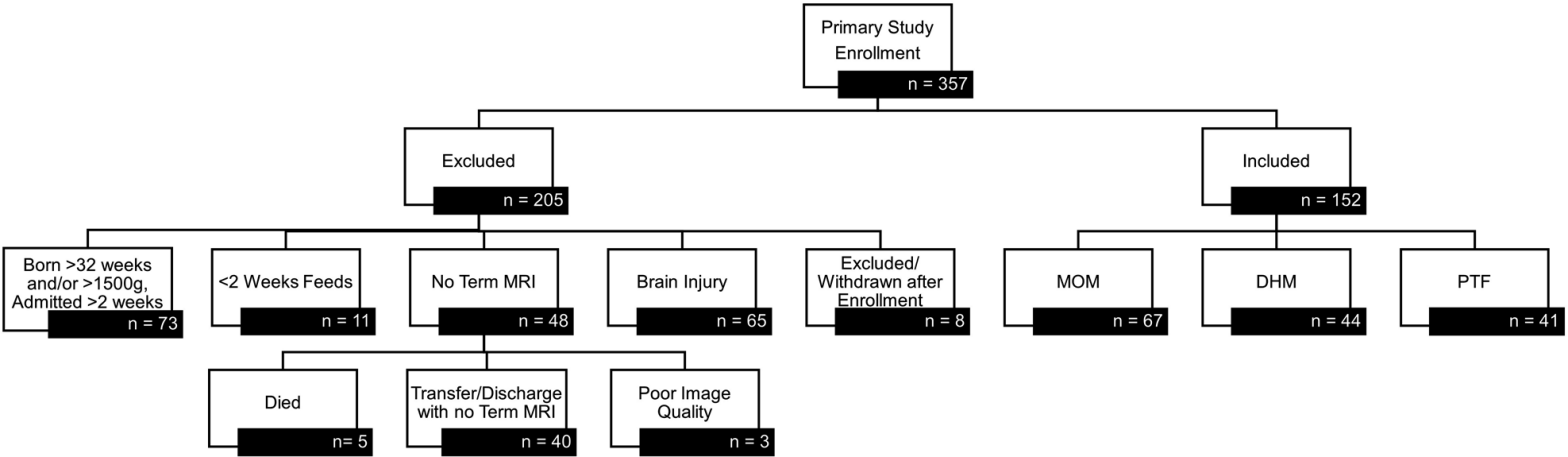
Patient exclusion and inclusion from primary observational cohort study for this secondary analysis. MOM mother’s own milk, DHM donor human milk, PTF preterm formula.

From the primary observational cohort study of 357 infants, 152 very preterm infants were eligible for this secondary analysis (67 mother’s own milk, 44 donor human milk, 41 preterm formula) (Fig. 2) with mean birth gestational age of 27.6 ± 2.5 weeks, mean birthweight of 976 ± 326 grams, and a female predominance (*n* = 90 [59%]) (Table 1). Eighteen infants in the primarily formula-fed group were born after donor milk was available. There were no differences in patient characteristics among infants excluded due to poor MRI image quality (*n* = 3). Systemic steroid exposure was highest in donor milk-fed infants (maternal milk 27%, donor milk 48%, preterm formula 39%, *p* = 0.07). There were no significant differences in other neonatal or maternal characteristics based on the type of enteral nutrition, including necrotizing enterocolitis.

**Table 1:** Patient characteristics by enteral nutrition type.

	Total (*n* = 152)	MOM (*n* = 67)	DHM (*n* = 44)	PTF(*n* = 41)	p*
Maternal age	28 (6)	29 (7)	29 (6)	27 (6)	0.14
Social Vulnerability Index, median (IQR)	0.7 (0.4, 0.9)	0.6 (0.4, 0.8)	0.7 (0.4, 0.9)	0.8 (0.5, 0.9)	0.37
Distance from hospital (miles), median (IQR)	12 (6, 32)	16 (7, 35)	15 (6, 46)	9 (6, 14)	0.63
Infant Race/Ethnicity, n (%)					0.14
Asian	20 (13)	10 (15)	8 (18)	2 (5)	
Black or African American	80 (53)	28 (42)	23 (52)	29 (71)	
Hispanic or Latino	19 (12)	12 (18)	4 (9)	3 (7)	
Native Hawaiian/Pacific Islander	6 (4)	3 (4)	2 (5)	1 (2)	
White	14 (9)	7 (10)	2 (5)	5 (12)	
Other/Unknown	13 (9)	7 (10)	5 (11)	1 (2)	
Antenatal corticosteroids, n (%)	82 (54)	40 (60)	22 (50)	20 (49)	0.45
Male sex, n (%)	62 (41)	30 (45)	18 (41)	14 (34)	0.56
Birth gestational age (weeks), mean (SD)	27.6 (2.5)	27.9 (2.4)	26.8 (2.2)	27.9 (2.7)	0.71
Discharge PMA (weeks), median (IQR)	39.1 (37, 41.4)	39 (36.9, 41.8)	39.5 (37, 40.6)	39.1 (37.4, 42.1)	0.79
Surgical necrotizing enterocolitis or SIP, n (%)	17 (11)	8 (12)	4 (9)	5 (12)	0.90
Systemic Steroids, n (%)	55 (36)	18 (27)	21 (48)	16 (39)	0.07
Bronchopulmonary Dysplasia, n (%)	75 (49)	31 (46)	22 (50)	22 (54)	0.75
Infection, n (%)	93 (61)	36 (54)	28 (64)	29 (71)	0.20
Retinopathy of Prematurity ≥ Stage 2, n (%)	45 (30)	17 (25)	13 (30)	15 (37)	0.46
Patent Ductus Arteriosus, n (%)	66 (43)	24 (36)	20 (45)	22 (54)	0.18
IVH, n (%)	46 (30)	24 (36)	19 (43)	13 (32)	0.87
Grade I	15 (10)	12 (18)	9 (20)	4 (10)	
Grade II	31 (20)	12 (18)	10 (23)	9 (22)	
Brain MRI PMA (weeks), mean (SD)	40 (2)	40.1 (1.9)	39.6 (1.9)	40.2 (2.2)	0.88
White matter injury score,^#^ median (IQR)	0 (0, 1)	0 (0, 1)	0 (0, 1)	0 (0,0)	0.46
*Growth & Nutrition*					
Birth weight (grams), mean (SD)	976 (326)	1033 (324)	899 (303)	966 (340)	0.20
Birth weight z-score, mean (SD)	–0.27 (0.91)	–0.24 (0.96)	–0.27 (0.80)	–0.33 (0.97)	0.88
Growth faltering, n (%)	94 (62)	40 (60)	32 (73)	22 (54)	0.17
Parenteral nutrition days, median (IQR)	20 (9, 39)	18 (10, 39)	25 (9, 38)	21 (11, 39)	0.52
Total nutrition days, mean (SD)	74 (26)	71 (24)	76 (25)	75 (28)	0.31
Percent enteral intake, median (IQR)					
% MOM	23 (3, 98)	100 (84, 100)	1 (0, 11)	5 (0, 16)	<0.001
% DHM	0 (0, 56)	0 (0, 0)	67 (59, 83)	0 (0, 14)	<0.001
% PTF	23 (0, 51)	0 (0, 13)	28 (9, 37)	91 (62, 99)	<0.001

^*^Analysis of Variance/Kruskal-Wallis Tests for continuous data, Chi-square/Fisher’s Exact Tests for categorical data.

^#^Kidokoro score for white matter cystic lesions or focal signal abnormality (range 0–7).

*MOM* Mother’s own milk, *DHM* donor human milk, *PMA* postmenstrual age, *PTF* preterm formula, *SIP* spontaneous intestinal perforation.

### Brain volumes

Term-equivalent volumetric data were available for all 152 infants (67 mother’s own milk, 44 donor human milk, 41 preterm formula). Intra- and inter-rater reliability intraclass correlation coefficients for MRI volumetric segmentation were 0.96 and 0.95, respectively. Total and tissue-specific brain volumes were lower in infants receiving donor human milk and preterm formula compared to mother’s own milk (Table 2). There were no significant differences in brain volumes between infants receiving donor human milk and mother’s own milk after adjusting for gestational age at birth and postmenstrual age at MRI. A sensitivity analysis did not reveal any significant differences in brain volumes between infants who received >70% cumulative enteral nutrition from donor human milk (*n* = 17) compared to mother’s own milk (*n* = 62).

**Table 2:** Brain volumes by enteral nutrition type.

					Comparison to MOM			
	Brain volume (cm^3^)^a^ Mean (SD)				DHM		PTF	
Brain Region	MOM (*n* = 67)	DHM (*n* = 44)	PTF (*n* = 41)		β (95% CI)^b^	*p*	Β (95% CI)^b^	*p*
Total Brain Volume	324.5 (46.7)	303.7 (43.7)		308.6 (55.2)	–5.9 (–19.9, 8.1)	0.40	–17.2 (–31.1, –3.3)	0.016*
Cortical Gray Matter	128.1 (23.6)	118.6 (23.4)		121.5 (27.9)	–1.7 (–8, 4.6)	0.56	–7.0 (–13.2, –0.8)	0.028
White Matter	144.6 (17.9)	135.7 (17.5)		137 (22.7)	–3.8 (–10.6, 2.9)	0.26	–7.6 (–14.3, –0.9)	0.026
Deep Gray Matter	24.2 (2.7)	23 (3.2)		23.1 (3.7)	–0.5 (–1.5, 0.6)	0.39	–1.2 (–2.2, –0.2)	0.024*
Amygdala-Hippocampus	2.3 (0.4)	2.2 (0.4)		2.2 (0.5)	–0.0 (–0.2, 0.1)	0.79	–0.1 (–0.2, 0.1)	0.21
Cerebellum	20.9 (4.8)	19.1 (5)		19.7 (5.2)	–0.3 (–1.8, 1.1)	0.65	–1.3 (–2.8, 0.1)	0.06
Brainstem	5.5 (0.8)	5.1 (0.7)		5.1 (0.6)	–0.2 (–0.4, 0.1)	0.16	–0.4 (–0.6, –0.2)	0.002*

^*^Significant *p* value < 0.025.

^a^Unadjusted mean.

^b^Generalized linear model adjusting for gestational age at birth and postmenstrual age at term-equivalent MRI with MOM as reference group.

*MOM* Mother’s own milk, *DHM* donor human milk, *PTF* preterm formula.

Infants receiving preterm formula demonstrated significantly lower brain volumes compared to mother’s own milk in the total brain (308.6 ± 55.2 vs. 324.5 ± 46.7 cm^3^, β = –17.2, *p* = 0.016), deep gray matter (23.1 ± 3.7 vs. 24.2 ± 2.7 cm^3^, β = –1.2, *p* = 0.024) and brainstem (5.1 ± 0.6 vs. 5.5 ± 0.8 cm^3^, β = –0.4, *p* = 0.002); volumes were also lower in the cortical gray matter (121.5 ± 27.9 vs. 128.1 ± 23.6 cm^3^, β = –7.0, *p* = 0.028) and white matter (137 ± 22.7 vs. 144.6 ± 17.9 cm^3^, β = –7.6, *p* = 0.026) compared to mother’s own milk. Incorporation of SVI into multivariate models yielded similar results except that the differences in cortical gray matter were more pronounced between preterm formula and maternal milk-fed infants (β = –7.4, *p* = 0.025), whereas differences in deep gray matter volumes (β = –1.2, *p* = 0.027) were no longer statistically significant (Supplementary Table S1). Adjusting for systemic steroid exposure did not significantly affect the relationship between preterm formula and maternal milk-fed infants in the brainstem (β = –0.3, *p* = 0.005) but attenuated the differences in total brain (β = –15.9, *p* = 0.03) and deep gray matter (β = –1.1, *p* = 0.04) volumes. Infant sex revealed no significant effects. In post-hoc analyses, brain volumes did not significantly differ between preterm formula compared to donor milk-fed infants (Table 5).

### White matter microstructure

**Fig. 3:**
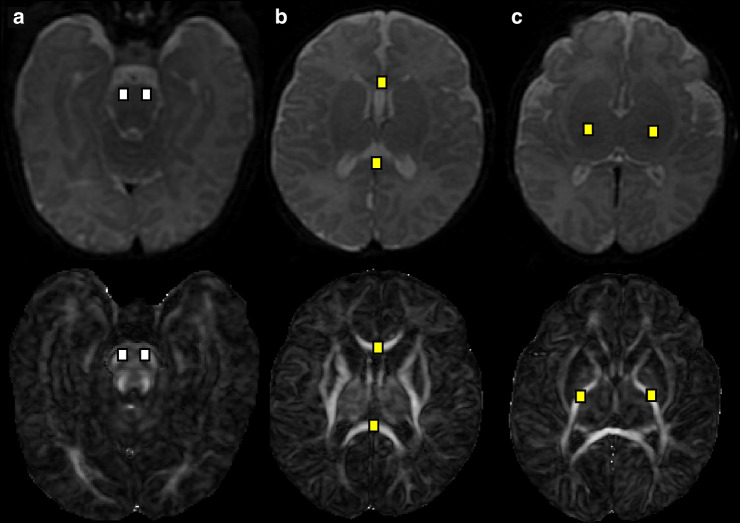
Term-equivalent axial DTI B0 images and parametric maps with white matter tracts demonstrating significant differences between maternal milk and donor human milk (white) or preterm formula-fed (yellow) infants. **a** Brainstem (pons), **b** corpus callosum, **c** posterior limb of internal capsule.

Term-equivalent DTI data were available for 146 (96%) infants (64 mother’s own milk, 43 donor human milk, 39 preterm formula); 6 were excluded due to poor DTI image quality. Intra- and inter-rater reliability intraclass correlation coefficients for ROI placement were 0.99 and 0.98, respectively. After adjusting for gestational age at birth and postmenstrual age at MRI, infants receiving donor human milk demonstrated lower MD in the brainstem (pons) compared to mother’s own milk (right: 0.78 ± 0.06 vs. 0.81 ± 0.08 mm^2^/second x10^–3^, β = –0.04, *p* = 0.008; left: 0.78 ± 0.07 vs. 0.82 ± 0.09 mm^2^/second x10^–3^, β = –0.06, *p* < 0.001) (Table 3) (Fig. 3a), with no significant differences between groups in FA (Table 4). A sensitivity analysis comparing infants who received >70% cumulative enteral nutrition from donor human milk (*n* = 17) to mother’s own milk (*n* = 62) also revealed lower MD in the brainstem (pons) of donor human milk-fed infants (right: β = –0.06, *p* = 0.018; left: β = –0.06, *p* 0.008).

**Table 3:** Diffusion tensor imaging mean diffusivity by enteral nutrition type.

				Comparison to MOM			
	Mean Diffusivity (x10^–3^ mm^2^/second)^a^			DHM		PTF	
Region of Interest	MOM (*n* = 64)	DHM (*n* = 43)	PTF (*n* = 39)	β (95% CI)^b^	*p*	β (95% CI)^b^	*p*
Corpus Callosum							
Genu	1.22 (0.22)	1.20 (0.22)	1.32 (0.18)	–0.05 (–0.1, 0.03)	0.23	0.11 (0.03, 0.19)	0.007*
Splenium	1.14 (0.22)	1.14 (0.25)	1.28 (0.28)	–0.01 (–0.1, 0.08)	0.75	0.13 (0.04, 0.22)	0.007*
Posterior Limb of Internal Capsule							
Right	0.91 (0.1)	0.89 (0.08)	0.96 (0.1)	–0.03 (–0.06, 0.01)	0.14	0.06 (0.02, 0.09)	0.002*
Left	0.91 (0.1)	0.9 (0.07)	0.97 (0.1)	–0.03 (–0.06, 0.01)	0.17	0.06 (0.03, 0.1)	0.001*
Brainstem - Pons							
Right	0.81 (0.08)	0.78 (0.06)	0.83 (0.10)	–0.04 (–0.07, –0.01)	0.008*	0.02 (–0.01, 0.05)	0.23
Left	0.82 (0.09)	0.78 (0.07)	0.82 (0.09)	–0.06 (–0.09, –0.03)	<0.001*	0.00 (–0.03, 0.03)	0.89

^*^Significant *p* value <0.025.

^a^Unadjusted mean.

^b^Generalized linear model adjusting for gestational age at birth and postmenstrual age at term-equivalent MRI with MOM as reference group.

*MOM* Mother’s own milk, *DHM* donor human milk, *PTF* Preterm formula.

**Table 4:** Diffusion tensor imaging fractional anisotropy by enteral nutrition type.

				Comparison to MOM			
	Fractional Anisotropy^a^			DHM		PTF	
Region of Interest	MOM (*n* = 64)	DHM (*n* = 43)	PTF (*n* = 39)	β (95% CI)^b^	*p*	β (95% CI)^b^	*p*
Corpus Callosum							
Genu	0.55 (0.09)	0.53 (0.11)	0.55 (0.10)	0.00 (0.–0.03, 0.04)	78	–0.01 (–0.04, 0.03)	0.64
Splenium	0.64 (0.11)	0.62 (0.10)	0.61 (0.11)	0.00 (–0.04, 0.04)	0.92	–0.03 (–0.07, 0.01)	0.19
Posterior Limb of Internal Capsule							
Right	0.62 (0.06)	0.61 (0.05)	0.59 (0.06)	–0.00 (–0.02, 0.02)	0.99	–0.02 (-0.05, –0.00)	0.019*
Left	0.60 (0.06)	0.60 (0.06)	0.59 (0.05)	0.00 (–0.02, 0.02)	0.96	–0.02 (–0.04, 0.00)	0.09
Brainstem - Pons							
Right	0.36 (0.07)	0.34 (0.07)	0.39 (0.1)	–0.01 (–0.04, 0.02)	0.63	0.03 (0.00, 0.06)	0.03
Left	0.37 (0.08)	0.34 (0.07)	0.39 (0.1)	–0.02 (–0.05, 0.01)	0.18	0.02 (–0.01, 0.05)	0.24

^*^Significant *p* value <0.025.

^a^Unadjusted mean.

^b^Generalized linear model adjusting for gestational age at birth and postmenstrual age at term-equivalent MRI with MOM as reference group.

*MOM* Mother’s own milk, *DHM* donor human milk, *PTF* preterm formula.

Infants receiving preterm formula demonstrated higher MD compared to mother’s own milk in the corpus callosum (genu: 1.32 ± 0.18 vs. 1.22 ± 0.22 mm^2^/second x10^–3^, β = 0.11, *p* = 0.007; splenium: 1.28 ± 0.28 vs. 1.14 ± 0.22 mm^2^/second x10^–3^, β = 0.13, *p* = .007) (Fig. 3b) and posterior limb of internal capsule (right: 0.96 ± 0.1 vs. 0.91 ± 0.1 mm^2^/second x10^–3^, β = 0.06, *p* = 0.002; left: 0.97 ± 0.1 vs. 0.91 ± 0.1 mm^2^/second x10^–3^, β = 0.06, *p* = 0.001) (Fig. 3c), and lower FA in the right posterior limb of internal capsule (0.59 ± 0.06 vs. 0.62 ± 0.06, β = –0.02, *p* = 0.019). Adjusting for systemic steroid exposure attenuated this FA difference in the right posterior limb of internal capsule (β = 0.024, *p* = 0.025) with no significant effects on MD (Supplementary Tables S2 and S3). Incorporation of SVI and infant sex into multivariate models revealed no significant effects. In post-hoc analyses, preterm formula-fed infants demonstrated significantly lower MD than donor milk-fed infants in all regions of interest, with significantly lower FA in the brainstem (pons) (right: β = –0.04, *p* = 0.018, left: β = –0.04, *p* = 0.025) (Table 5).

**Table 5:** Brain volumes and diffusion tensor imaging between donor human milk and formula-fed infants.

Brain Volumes				Diffusion Tensor Imaging			
				Mean Diffusivity (x10^−3^ mm^2^/second)		Fractional Anisotropy	
Brain region	β (95% CI)^a^	*p*	Brain Region	β (95% CI)^a^	*p*	β (95% CI)^a^	*p*
Total Brain Volume	10.2 (–7.0, 27.3)	0.24	Corpus Callosum Genu Splenium	–0.17 (–0.25, –0.08) –0.15 (–0.26, –0.04)	<0.001* 0.010*	(–0.03, 0.06) 0.03 (–0.02, 0.07)	0.50 0.26
Cortical Gray Matter	4.9 (–2.7, 12.4)	0.20					
White Matter	3.5 (–4.8, 11.8)	0.40	Posterior Limb of Internal Capsule Right Left	–0.08 (–0.12, –0.05) –0.09 (–0.13, –0.05)	<0.001* <0.001*	(0.00, 0.05) 0.02 (-0.01, 0.04)	0.07 0.16
Deep Gray Matter	0.7 (–0.7, 2.0)	0.34					
Amygdala-Hippocampus	0.1 (–0.1, 0.2)	0.44					
Cerebellum	1.0 (–0.8, 2.7)	0.29	Brainstem - Pons Right Left	–0.06 (–0.1, –0.03) –0.06 (–0.09, –0.03)	<0.001* <0.001*	–0.04 (–0.08, –0.01) –0.04 (–0.07, –0.01)	0.018* 0.025
Brainstem	0.2 (–0.1, 0.4)	0.12					

^*^Significant *p* value <0.025.

^a^Generalized linear model adjusting for gestational age at birth and postmenstrual age at term-equivalent MRI with PTF as reference group.

## Discussion

Consistent with our hypothesis, very preterm infants supported with primarily mother’s own milk, donor human milk, or preterm formula during the *ex-utero* third trimester demonstrated distinct patterns of structural brain development using quantitative MRI at TEA. Brain volumes did not significantly differ between infants supported with donor human milk and mother’s own milk, while preterm formula-fed infants demonstrated lower total and tissue-specific brain volumes in the deep gray matter and brainstem. DTI-based microarchitectural brain analyses also revealed differences in white matter microstructural organization between donor human milk and preterm formula-fed infants compared to those receiving mother’s own milk, with greater alterations noted in those receiving preterm formula. Direct comparisons between donor human milk and preterm formula-fed infants suggested more advanced white matter microstructural organization in those receiving donor human milk, with no significant differences in brain volumes between groups.

Existing MRI studies relating human milk feeding to preterm brain development at TEA do not make a distinction between maternal and donor human milk.^9,28,33^ Pasteurized donor human milk differs in composition from raw preterm maternal milk due to both its source and processing.^17–19,42–44^ Donated milk most often originates from mothers of term infants with an established milk supply, representing a later stage of lactation with lower macronutrient and energy content compared to early preterm maternal milk.^45^ It then undergoes processing, including pasteurization and multiple freeze-thaw cycles, that further decreases macronutrient levels and destroys or diminishes many bioactive factors.^17–19^ Despite these differences, our findings suggest that nutritional support with donor human milk is potentially advantageous over preterm formula in promoting *ex-utero* third trimester brain development in very preterm infants when mother’s own milk is unavailable.

We report a positive association between maternal milk intake and term-equivalent brain volumes in very preterm infants, with maternal milk-fed infants demonstrating larger total brain volumes as well as greater tissue-specific volumes in the deep gray matter and brainstem compared to formula-fed infants. Prior studies also note larger gray matter volume in maternal milk compared to formula-fed preterm infants.^12,31^ In a similar cohort of very preterm infants, early maternal milk intake was positively associated with term-equivalent deep gray matter volume and correlated with improved intelligence quotient, motor function, and school-related performance on mathematics and working memory at 7 years of age.^12^ Although a connection between maternal milk intake and brainstem volume has not been previously described, term-equivalent MRI studies have established the brainstem as a nutritionally sensitive region, with volumetric growth predicting neurodevelopmental outcomes through early school age.^46–48^ Importantly, we did not find any significant differences in brain volumes between donor and maternal-milk fed infants even after sensitivity analyses using a higher threshold ( >70%) of cumulative enteral intake, suggesting that at least some of the factors mediating the positive effect of a human milk diet on *ex-utero* preterm brain development are preserved with pasteurized donor milk feeding.

DTI measures in our cohort were consistent with more advanced white matter microstructural organization in the corpus callosum and posterior limb of internal capsule in maternal and donor milk compared to preterm formula-fed infants, where white matter development is characterized by decreasing MD and increasing FA as a function of advancing gestational age.^25,49^ Duration of human milk feeding in preterm infants has been linked to improved organization of the corpus callosum by TEA, a marker of cognitive and motor outcomes through school age.^25,32,50,51^ Posterior limb of internal capsule development in preterm infants by term age also correlates with later neurodevelopment.^25,52^ Lower FA and higher MD, as seen in our formula-fed cohort, correspond to lower cognitive and motor scores through 2 years of age.^25,52^ It is challenging to interpret the differences in brainstem MD noted between maternal and donor human-milk fed infants in our study cohort, as the normal evolution of diffusion-based measures for brainstem white matter tracts have not been well-described. Limited DTI investigations of neonatal brainstem white matter development suggest that MD may progress in an opposite direction from the supratentorial white matter and increase with advancing gestational age, such that the lower values in our donor human milk-fed infants may represent less advanced maturation.^41,53^ Taken together, our DTI findings suggest differences in white matter microstructural organization in both donor human milk and preterm formula-fed infants compared to maternal milk, with more striking differences noted in those receiving preterm formula.

Randomized-controlled trials have not observed benefits of nutrient-fortified, pasteurized donor human milk feeding over preterm formula through toddler age in very preterm infants using traditional neurodevelopmental assessments. O’Connor et al. did not find significant differences in 18-month Bayley Scales of Infant and Toddler Development (BSID-III) scores between very low birth weight ( <1500 g) infants receiving maternal milk supplemented with either donor milk or preterm formula.^22^ Both donor milk and formula-supplemented infants in this study received a high proportion of maternal milk. To evaluate the impact of a donor milk diet on preterm neurodevelopment, Colaizy et al. randomized extremely preterm infants receiving minimal maternal milk to a primary diet of either donor human milk or preterm formula.^23^ BSID-III scores in this study also did not differ significantly between groups at 22-26 months’ corrected age. Although findings in our cohort suggest a benefit of donor milk compared to formula feeding for *ex-utero* structural brain development by TEA, infants receiving minimal maternal milk represent a particularly at-risk population with coexisting vulnerabilities that could adversely impact neurodevelopment and lead to post-discharge attenuation of beneficial donor milk effects.^12,54^ It is also possible that these neuroimaging findings may correlate to more subtle learning and behavioral differences that have either not yet emerged by toddler age or are not adequately detected by the BSID-III assessment. Additional studies are needed to better elucidate the long-term neurobehavioral outcomes in donor milk-fed preterm infants through school age. Further investigation is also necessary to establish thresholds of donor milk exposure for optimal neurodevelopmental outcomes. Neurodevelopmental follow-up in our cohort is underway to assess the long-term implications of our findings.

Although our study has several strengths, our conclusions are limited by the observational nature of our cohort. Infants were not randomized to type of enteral nutrition. It also is possible for residual confounding from covariates not assessed in our analysis that could account for differences in infants receiving mother’s own milk, donor human milk, or preterm formula. Our analyses were also limited by how the groups were defined, as a threshold of >50% still permits exposure to other enteral nutrition types. Infants receiving primarily donor human milk had the greatest exposure to other enteral nutrition types with an average 67% intake, which may have blunted differences. We also did not have complete data on additional individual-level markers of socioeconomic status and factors known to affect mother’s ability to provide milk as well as brain development, such as parental education or household income.^55,56^ Though SVI scores in our cohort were not significantly different between enteral feeding groups, maternal milk-fed infants had lower scores than formula-fed infants, reflecting lower overall social vulnerability. Given that incorporation of SVI into our statistical model attenuated the difference in deep gray matter volume between these two groups, additional work is needed to better understand the social constraints that may hamper the ability to provide mother’s own milk.

## Conclusions

This study affirms mother’s own milk as the gold standard for very preterm infant enteral nutrition and suggests that donor human milk may be advantageous over preterm formula for promoting *ex-utero* third trimester brain structure in the absence of sufficient maternal milk. Brain volumes were not significantly different between maternal and donor human milk-fed infants. In contrast, infants receiving preterm formula demonstrated significantly lower total and tissue-specific brain volumes and altered white matter microstructural organization in multiple early developing white matter tracts compared to maternal milk-fed infants. These findings lend support to the current clinical practice of supplementation with donor human milk when maternal milk is unavailable and emphasize the importance of promoting mother’s own milk for all very preterm infants.

## Supplementary information

Supplementary Tables

## Data Availability

The datasets generated during and/or analyzed during the current study are available from the corresponding author on reasonable request.
