# Supplementary material for: Donor human milk and structural brain development in very preterm infants
Source: Pediatr Res. 2025 Nov 1;99(7):2640–8. doi: 10.1038/s41390-025-04539-3 (PMC12959636; doi:10.1038/s41390-025-04539-3)
Supplement: Supplementary file 1 [file 41390_2025_4539_MOESM1_ESM.pdf]

Supplementary Table S1. Generalized linear models of brain volumes by enteral nutrition type

Brain Region	Model 1[a] (Birth GA + MRI PMA)				Model 2[a] (Birth GA + MRI PMA + SVI)				Model 3[a] (Birth GA + MRI PMA + Steroids)			
	DHM		PTF		DHM		PTF		DHM		PTF	
	β (95% CI)	*p*	β (95% CI)	*p*	β (95% CI)	*p*	β (95% CI)	*p*	β (95% CI)	*p*	β (95% CI)	*p*
Total Brain Volume	-5.9 (-19.9, 8.1)	.40	-17.2 (-31.1, -3.3)	**.016***	-6.2 (-20.4, 8.1)	.39	-17.8 (-32.2, -3.3)	**.017***	-4.8 (-18.7, 9.2)	.50	-15.9 (-29.8. -1.9)	.03
Cortical Gray Matter	-1.7 (-8, 4.6)	.56	-7.0 (-13.2, -0.8)	.028	-1.9 (-8.3, 4.5)	.55	-7.4 (-13.9, -0.9)	.025	-1.1 (-7.4, 5.1)	.72	-6.4 (-12.6, -0.1)	.05
White Matter	-3.8 (-10.6, 2.9)	.26	-7.6 (-14.3, -0.9)	.026	-3.8 (-10.7, 3.1)	.28	-7.7 (-14.6, -0.7)	.031	-3.6 (-10.4, 3.2)	0.30	-7.4 (-14.1, -0.6)	.03
Deep Gray Matter	-0.5 (-1.5, 0.6)	.39	-1.2 (-2.2, -0.2)	**.024***	-0.5 (-1.5, 0.6)	.40	-1.2 (-2.3, -0.1)	.027	-0.3 (-1.4, 0.7)	0.51	-1.1 (-2.1, -0.0)	.04
Amygdala-Hippocampus	-0.0 (-0.2, 0.1)	.79	-0.1 (-0.2, 0.1)	.21	-0.0 (-0.2, 0.1)	.77	-0.1 (-0.3, 0.1)	.21	-0.0 (-0.2, 0.1)	0.82	-0.1 (-0.2, 0.1)	.23
Cerebellum	-0.3 (-1.8, 1.1)	.65	-1.3 (-2.8, 0.1)	.06	-0.3 (-1.8, 1.2)	.69	-1.3 (-2.8, 0.2)	.08	-0.1 (-1.5, 1.3)	0.89	-1.1 (-2.5, 0.3)	.32
Brainstem	-0.2 (-0.4, 0.1)	.16	-0.4 (-0.6, -0.2)	**.002***	-0.2 (-0.4, 0.1)	.20	-0.4 (-0.6, -0.1)	**.002***	-0.1 (-0.4, 0.1)	0.29	-0.3 (-0.6, -0.1)	**.005***

DHM: Donor human milk, GA: Gestational age, PMA: Postmenstrual age, PTF: Preterm formula, SVI: Social vulnerability index

*Significant p-value <.025

[a]Reference group for analyses is mother's own milk

Supplementary Table S2. Generalized linear models of diffusion tensor imaging mean diffusivity by enteral nutrition type

**ROI**	**Model 1[a] *(Birth GA + MRI PMA)***				**Model 2[a] *(Birth GA + MRI PMA + SVI)***				**Model 3[a] *(Birth GA + MRI PMA + Steroids)***			
	**DHM**		**PTF**		**DHM**		**PTF**		**DHM**		**PTF**	
	B **(95% CI)**	***p***	β **(95% CI)**	***p***	β **(95% CI)**	***p***	β **(95% CI)**	***p***	β **(95% CI)**	***p***	β **(95% CI)**	***p***
CC												
Genu	-0.05(-0.1,0.03)	.23	0.11(0.03,0.19)	**.007***	-0.05(-0.1,0.03)	.22	0.11(0.03,0.19)	**.010***	-0.05(-0.1,0.03)	.20	0.11(0.03,0.19)	**.009***
Splenium	-0.01(-0.1,0.08)	.75	0.13(0.04,0.22)	**.007***	-0.02(-0.1,0.08)	.75	0.13(0.04,0.22)	**.008***	-0.02(-0.1,0.07)	.66	0.12(0.04,0.22)	**.010***
PLIC												
Right	-0.03(-0.06,0.01)	.14	0.06(0.02,0.09)	**.002***	-0.03(-0.07,0.01)	.10	0.05(0.02,0.09)	**.006***	-0.03(-0.06,0.01)	.13	0.06(0.02,0.09)	**.003***
Left	-0.03(-0.06,0.01)	.17	0.06(0.03,0.1)	**.001***	-0.03(-0.07,0.01)	.10	0.06(0.03,0.2)	**.004***	-0.03(-0.06,0.01)	.19	0.06(0.03,0.10)	**.001***
Pons												
Right	-0.04(-0.07,-0.01)	**.008***	0.02(-0.01 0.05)	.23	-0.04(-0.07,-0.01)	**.011***	0.02(-0.01 0.05)	.23	-0.04(-0.07,-0.01)	**.009**	0.02(-0.01 0.05)	.23
Left	-0.06(-0.09,-0.03)	**<.001***	0.00(-0.03,0.03)	.89	-0.05(-0.08,-0.02)	**<.001***	0.00(-0.03,0.03)	.89	-0.06(-0.09,-0.03)	**<.001***	0.00(-0.03,0.03)	.96

CC: Corpus callosum, DHM: Donor human milk, GA: Gestational age, PLIC: Posterior limb of internal capsule, PMA: Postmenstrual age, PTF: Preterm formula, ROI: Region of interest, SVI: Social Vulnerability Index

*Significant p-value <.025

[a]Reference group for analyses is mother's own milk

Supplementary Table S3. Generalized linear models of diffusion tensor imaging fractional anisotropy by enteral nutrition type

ROI	Model 1[a] (Birth GA + MRI PMA)				Model 2[a] (Birth GA + MRI PMA + SVI)				Model 3[a] (Birth GA + MRI PMA + Steroids)			
	DHM		PTF		DHM		PTF		DHM		PTF	
	B (95% CI)	*p*	B (95% CI)	*p*	β (95% CI)	*p*	B (95% CI)	*p*	β (95% CI)	*p*	β (95% CI)	*p*
CC												
Genu	0.00(0.-0.03,0.04)	.78	-0.01(-0.04,0.03)	.64	0.00(-0.03,0.04)	.81	-0.01(-0.04,0.03)	.62	0.01(-0.02,0.04)	.51	-0.00(-0.04,0.03)	.88
Splenium	0.00(-0.04,0.04)	.92	-0.03(-0.07,0.01)	.19	0.00(-0.04,0.04)	.95	-0.03(-0.07,0.01)	.17	0.01(-0.03,0.05)	.63	-0.02(-0.06,0.02)	.31
PLIC												
Right	-0.00(-0.02,0.02)	.99	-0.02(-0.05,-0.00)	**.019***	-0.00(0.02,0.03)	.95	-0.03(-0.05,-0.00)	**.018***	0.00(-0.02,0.02)	.10	-0.02(-0.05,-0.00)	**.025**
Left	0.00(-0.02,0.02)	.96	-0.02(-0.04,0.00)	.09	0.00(-0.02,0.03)	.63	-0.01(-0.03,0.00)	.27	0.00(-0.02,0.02)	.15	-0.02(-0.04,0.00)	.11
Pons												
Right	-0.01(-0.04,0.02)	.63	0.03(0.00,0.06)	.03	-0.00(-0.04,0.02)	.56	0.03(0.00,0.06)	.05	-0.01(-0.04,0.02)	.66	0.03(0.00,0.06)	.03
Left	-0.02(-0.05,0.01)	.18	0.02(-0.01,0.05)	.24	-0.02(-0.05,0.01)	.15	0.02(-0.02,0.05)	.32	-0.02(-0.05,0.01)	.21	0.02(-0.01,0.05)	.22

CC: Corpus callosum, DHM: Donor human milk, GA: Gestational age, PLIC: Posterior limb of internal capsule, PMA: Postmenstrual age, PTF: Preterm formula, ROI: Region of interest, SVI: Social Vulnerability Index

*Significant p-value <.025

[a]Reference group for analyses is mother's own milk
